# Solitary metastasis to the skin and colon from gastric cancer after curative gastrectomy and chemotherapy: A case report

**DOI:** 10.1097/MD.0000000000021532

**Published:** 2020-07-31

**Authors:** Shuai Yang, Xiang-Liang Liu, Xiang-Ling Guo, Bin Song, Shou-Zhen Li, Xiao-Feng Sun, Ye Feng

**Affiliations:** a Department of ultrasound; b Cancer Center, the First Hospital of Jilin University, Changchun, Jilin, China; c Department of Gastroenterology, Jilin Province People's Hospital; d Department of Gastrointestinal colorectal and anal surgery, China-Japan Union Hospital of Jilin University, Changchun, Jilin, China.

**Keywords:** case report, colonic metastasis, cutaneous metastasis, gastric cancer, poorly differentiated adenocarcinoma

## Abstract

**Rationale:**

Gastric cancer usually spread via blood circulation to liver, lung, bone, and kidney after recurrence, but it is extremely rare in clinical practice that gastric carcinoma metastasizes to the skin and colon without metastasis to common sites like liver or lung.

**Patient concerns:**

A 57-year-old man was admitted to the hospital with altered bowel habit and hematochezia for 2 weeks.

**Diagnoses:**

The patient was diagnosed with advanced gastric cancer at stage IIIA (pT3N2M0) two and a half years ago. Cutaneous metastasis from gastric cancer was confirmed by cutaneous biopsy 2 years following curative gastrectomy. Unfortunately, colonic metastasis from gastric cancer was found by PET-CT 6 months after the diagnosis of cutaneous metastasis.

**Interventions:**

The patient was given chemotherapy with docetaxel, cisplatin, and 5-fluorouracil for the skin metastasis. Right hemicolectomy was performed when the malignant tumor of the colon was found, in order to relieve the symptom, and improve the quality of life.

**Outcomes:**

The patient was treated with chemoradiotherapy in a local hospital, the peritoneal carcinomatosis occurred 5 months after the second operation, and died 9 months after the diagnosis of colonic metastasis.

**Lessons:**

Our case represents a rare condition that solitary cutaneous and colonic metastasis from gastric cancer can occur after surgical resection and systemic chemotherapy. Its unique clinicopathological features can extend our insights on gastric cancer, and it may provide clinicians with some positive clinical experience for identifying and treating this disease.

## 1 Introduction

Gastric cancer is the second cause of cancer-related mortality in China.^[1]^ Advanced gastric carcinoma usually metastasizes to the liver, regional lymph nodes, peritoneum, and lung. Skin metastasis from all gastric cancer only occurs in approximately 0.8% to 1.0% of gastric cancer patients.^[2]^ In 2.3% of gastric cancer patients, metastasizing to the intestinal tract could happen.^[3]^ Skin metastasis usually appears in 1 to 3 years, with a maximum of 15 years, after surgical resection.^[4]^ The common location of metastatic lesions is the abdominal wall, neck, back, and inguinal region, and these often present as single or multiple painless nodules with an erysipelas-like appearance.^[5]^ These lesions may be easily misdiagnosed as superficial soft connective tissue tumors, such as neurofibroma and liposarcoma.^[6]^ Colonic involvement usually occurs 16 months after the primary lesion was found.^[3]^ The most frequently involved sites for colonic metastasis are the transverse colon. Colonic metastasis from gastric cancer usually presents as an annular stricture, a linitis plastica-like appearance, or a polypoid lesion.^[7]^ It is extremely rare for gastric cancer to initially metastasize to the skin and subsequently metastasize to the right hemicolon, while it does not metastasize to the liver or other visceral organs after curative resection. The case study aims to present a kind of rare metastasis from gastric cancer to improve clinicians’ understanding of the biological behavior of gastric cancer.

## 2 Case report

**Figure 1:**
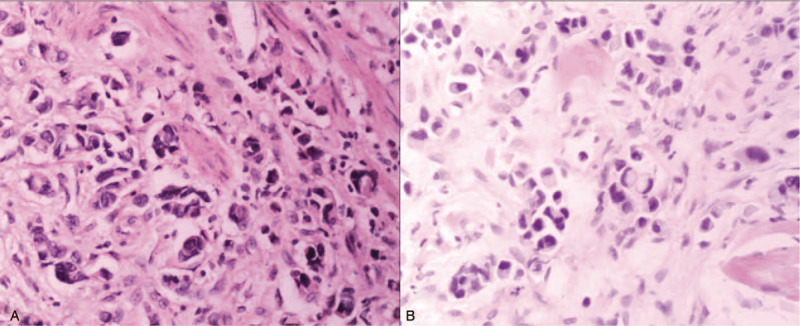
A: The poorly differentiated adenocarcinoma of the stomach characterized by signet-ring cell carcinoma and mucinous adenocarcinoma (H&E × 200); B: The infiltration of the skin by poorly differentiated adenocarcinoma that originated from gastric cancer (H&E × 200).

A 57-year-old man was referred to the China-Japan Union Hospital of Jilin University (Changchun, China) with altered bowel habit and hematochezia for two weeks in July 2017. About two and a half years before, the patient was diagnosed as gastric cancer and had no known past medical history and relevant family cancer history, and then the patient underwent distal gastrectomy with D2 lymphadenectomy, and the gastrointestinal continuity was built by Billroth II antecolic loop gastrojejunostomy. Histological examination of the resected tissue revealed poorly differentiated adenocarcinoma that involved signet-ring cell carcinoma and mucinous adenocarcinoma. The tumor invaded into the serosa with 2/12 and 4/16 lymph nodes of the lesser and greater curvatures of stomach positive for metastatic carcinoma respectively. Only lymphovascular invasion was noted and margins were clean. (Fig. 1A). The adenocarcinoma was classified as stage IIIA (T3N2M0), according to the 7^th^ edition of the American Joint Committee on Cancer (AJCC). Immunohistochemistry results revealed that it was positive for cytokeratin (CK)-7 and Ki67 (30%), and negative for epidermal growth factor receptor (EGFR), human epidermal growth factor receptor-2 (HER-2), CK20 and vimentin (Vim) (Fig. 4). The patient was given adjuvant chemotherapy with SOX (oxaliplatin, 130 mg/m^2^; S-1, 60 mg/day) for 6 cycles because of the poor postoperative staging classification. The patient recovered well after surgical resection and chemotherapy.

In January 2017, the patient presented with nape and shoulder pain, and clinical examination revealed a 10 × 12 cm irregular thickening of the subcutaneous tissue on the nape and bilateral scapular region. The patient's serum CEA level was 22.8 μg/L and cutaneous biopsy revealed a poorly differentiated adenocarcinoma (Fig. 1B). Immunohistological staining indicated that it was negative for CK20 and positive for CK7, Villin, caudal type homeobox transcription factor (CDX)-2, Cadherin, Ki67 (60%) and 1+ expression of HER-2 (Fig. 4). Morphology observation and immunohistochemistry results indicated that the cutaneous adenocarcinoma originated from the primary stomach adenocarcinoma. The patients refused to perform genetic testing because of poor economic condition and underwent another six cycles of chemotherapy with docetaxel (75 mg/m^2^/day), cisplatin (75 mg/m^2^/day) and 5-fluorouracil (1000 mg/m^2^/day) due to the recurrence and metastasis of gastric cancer, and the skin lesion was shrunk after adopting chemotherapy. However, the patient refused radiation therapy treatment due to the concern of radiation-induced myelopathy.

**Figure 2:**
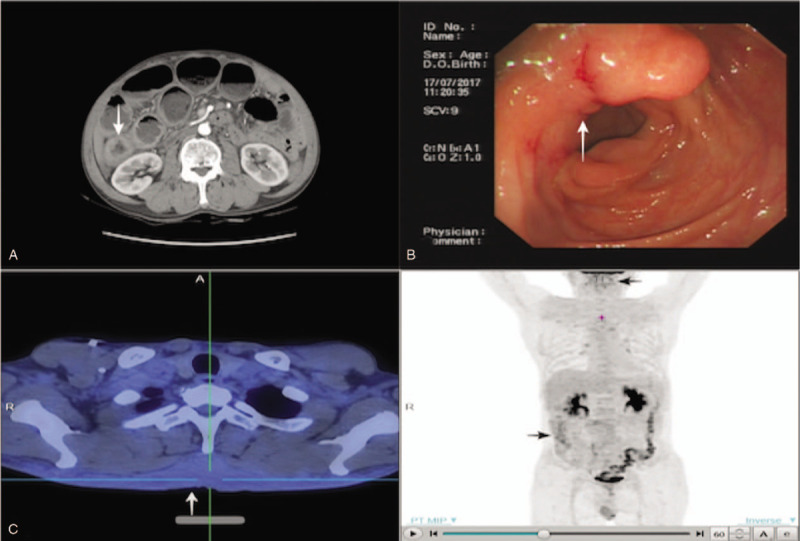
A: Abdominal CT revealed the lumen stenosis and the uneven thickening of the ascending colon and ileocecal region. B: Colonoscopy revealed that the surface of the ileocecal valves was rough and uneven, and the ascending colon presented as an annular stricture. C: FDG-PET/CT examination: This indicated a slightly abnormal 18F-FDG uptake at the adipose layer of the nape and bilateral scapular region, and the right hemicolon after chemotherapy.

**Figure 3:**
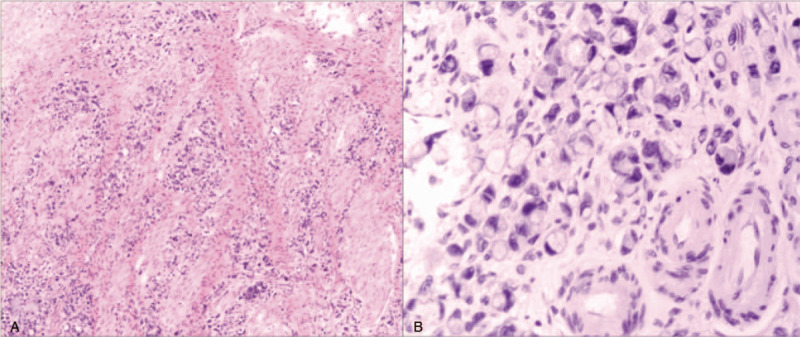
Histological examination of the colon cancers derived from gastric cancer metastasis. This revealed the poorly differentiated adenocarcinoma with signet ring cell carcinoma in the colon mucosa. A: low magnification of the colon tumor section (H&E × 40); B: high magnification of the colon tumor section (H&E × 200).

**Figure 4:**
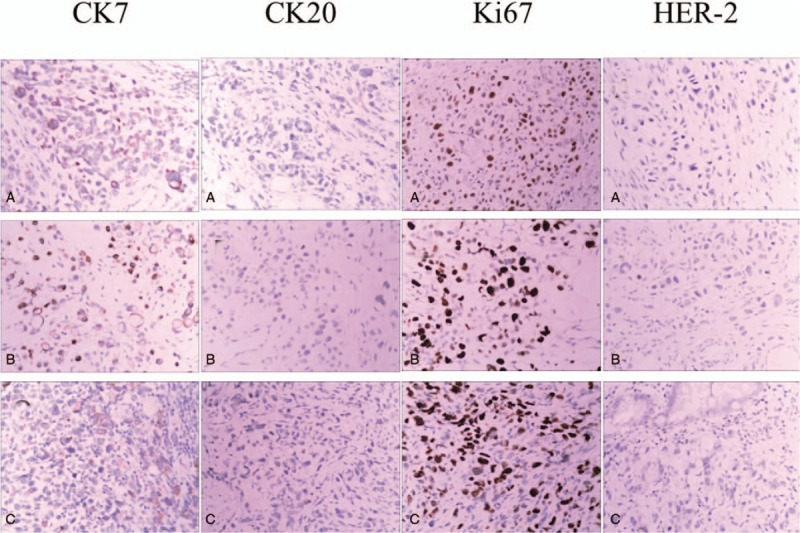
Immunohistochemistry of gastric tumors, skin tumors, and colon tumors. This revealed that both the primary site and metastatic sites have similar patterns of cytokeratin expression, but different Ki67 expression levels and HER-2 status. **A**: primary site; B: cutaneous metastasis; C: colonic metastasis. (all, H&E × 200).

The patient was well until he was referred to the hospital again in July 2017. Physical examination revealed that the patient had tenderness on the right lower abdomen without obvious anemic symptom or enlarged inguinal lymph nodes. Abdominal CT revealed lumen stenosis and uneven thickening of the ascending colon and ileocecal region (Fig. 2A). Colonoscopy revealed that the surface of the ileocecal valves was rough and uneven, and the ascending colon presented as an annular stricture (Fig. 2B). Subsequently, the FDG-PET/CT result indicated a slightly abnormal ^18^F-FDG uptake at the adipose layer of the nape and bilateral scapular region and the right hemicolon (Fig. 2C). Besides, the biopsy result revealed poorly differentiated adenocarcinoma. Meanwhile, the levels of serum tumor markers carbohydrate antigen (CA) 19–9, CA125, CA242, and CEA were significantly elevated to 642.55 U/ml, 38.02 U/ml, 109.20 U/ml and 21.80 ng/ml, respectively. An uneven mass was found in the ascending colon and ileocecal region by palpating and no peritoneal dissemination site was found by visual inspection during the operation. Thus, right hemicolectomy with mesenteric lymph node dissection was performed. Histological examination revealed a poorly differentiated adenocarcinoma with signet-ring cell carcinoma, the tumor infiltrated into the serosa and 15 of 19 lymph nodes were involved, lymphovascular and perineural invasion were noted, and margins were clean (Fig. 3). Immunohistochemistry results revealed that it was negative for CK20, focally positive for CK7 and positive for Villin, CDX-2, Cadherin17, Ki67 (60%), and 1+ expression of HER-2. Pathology results supported the diagnosis that the carcinoma in colon derived from the previous gastric carcinoma (Fig. 4). Following the second operation, the patient was transferred to the Department of Hematology and Oncology and received adjuvant chemoradiotherapy in a local hospital. However, the peritoneal carcinomatosis happened 5 months after the second operation, and the patient succumbed to the disease 9 months after the diagnosis of colonic metastasis.

## 3 Discussion

The accessory examination, colonoscopy, FDG-PET/CT, CT, and needle biopsy could provide clues to diagnose clinically rarely observed metastasis from gastric cancer to the skin and the intestinal tract.^[2,3]^ FDG-PET/CT has been considered as a valuable diagnostic method for advanced metastatic gastric cancer,^[8]^ but the metastatic lesions in our patient cannot be detected obviously by PET scan, the possible cause is that PET-CT assessment took place after the second chemotherapy, the result of PET scan evaluation may be influenced by chemotherapy due to the positive anti-tumor effect of chemotherapy. The tumor markers like CA19–9 and CEA are also good indicators for recurrence and metastasis of gastric cancer. Skin metastasis and colonic metastasis from gastric cancer were diagnosed with the result of accessory examination, tumor markers, and pathology.

The route for gastric cancer cells to spread to the skin and colon may be different. Lymphatic and hematogenous dissemination could be the possible route for distant skin metastasis, while direct invasion, hematogenous, lymphatic, and peritoneal seeding are traditionally considered as the routes for colonic metastasis.^[9]^ Besides, some studies have indicated that gastric cancer could metastasize to privileged sites through the fifth route, in which gastric cancer cells could drop into the “bare area” of the mesogastrium and subsequently scatter in the abdominal cavity during surgical resection, ultimately leading to locoregional recurrence.^[10]^ Since skin metastasis occurred before colonic metastasis, the colonic metastasis might derive from the skin lesion, but recent studies have shown that circulating tumor cells existing in peripheral blood is also link to recurrence and metastasis of gastric carcinoma,^[11]^ so the exact relationship between the skin metastasis and the colonic metastasis could not be determined due to limitation of detection technology, insufficient cognition of cancer and heterogeneity in the process of tumor progression. The mechanisms predisposing gastric cancer to metastasize to the skin and colon remain unclear. One possibility is that the skin or colon may provide a suitable microenvironment for a particular sub-clone of the tumor. It has been reported that some chemokines and their receptors participate in organ selective tumor metastasis. Chemokine CCL27, a member of the CC chemokines, is highly expressed in the skin. Its specific receptor CCR10 is involved in cutaneous metastasis of melanoma,^[12]^ and is also expressed in gastric cancer cells.^[13]^ Such findings indicate that CCL27/CCR10 may also play an important role in skin metastasis of gastric cancer, which needs to be further investigated.

In this case, the cutaneous and colonic metastasis that originated from the gastric cancer were poorly differentiated adenocarcinoma with signet-ring cell carcinoma, which is in agreement with the observations in the previous studies.^[9]^ The advanced histological grade and positive expression of ki67 status in the primary gastric cancer and gastric cancer metastasis-derived skin cancer and intestinal cancer implicated that the cancer was highly malignant, and would result in poor clinical outcome.^[14,15]^ HER-2 overexpression is closely associated with cell proliferation and differentiation and tumor invasion, and is an important predictor for the prognosis of gastric cancer.^[16]^ In the present case, the HER-2 levels in both primary sites and metastatic sites were considered as negative, according to present NCCN guidelines. However, HER-2 expression level appeared to be slightly higher in the metastatic sites than in the primary sites for this patient, which is consistent with the known results that HER-2 could be positively expressed in lymphatic metastasis lesions and distant metastasis lesions, while the primary tumors were HER-2 negative.^[17,18]^ It was proposed that the balance of polyclone in a tumor may break and lead to a particular clone of high metastatic potential,^[19]^ which may tend to migrate to different sites.

Cutaneous metastasis and colonic metastasis are indicative of the end-stage of gastric cancer with poor prognosis and short survival time. Most patients died within several months after the disease be diagnosed, especially patients with multiple metastases.^[20,21]^ Treatment for distant metastasis of gastric carcinoma usually consists of adjuvant chemotherapy and supportive care. Radiotherapy could exert a positive long-term impact on oligometastasis of gastric carcinoma according to recent researches.^[22,23]^ Furthermore, local radiotherapy may cause immunogenic death of tumor cells and tend to result in systemic anti-tumor effect mediated by the immune system.^[24]^ Whether the radiotherapy could make a positive difference in metastatic gastric carcinoma, especially the type of oligometastasis in the present case, is debatable. In some cases, surgical resection is necessary to control severe symptoms and ameliorate the quality of patients’ life.^[25]^ We considered the operation for the sake of relieving the symptoms of hematochezia and abdominal distention caused by lumen stenosis of colon and improving the quality of life as well. And with the development of molecular targeted therapies and immunotherapies, VEGFR-2 inhibitors and PD-1 antibodies could also provide a good choice for the treatment of gastric carcinoma with HER-2 negative status.^[26–28]^ The optimal management strategy of this kind of metastasis from gastric cancer still needs further investigation because of limited data.

## 4 Conclusion

We should be alert that the possibility of solitary metastasis to the skin and colon from gastric carcinoma after systemic therapy, PET scan and tumor markers may offer clues for the diagnosis of metastasis of gastric carcinoma. This case could offer positive clinical experience for physicians to identify and treat this disease.

## Author contributions

**Conceptualization:** Xiao-Feng Sun.

**Data curation:** Shuai Yang, Xiang-Ling Guo.

**Writing – original draft:** Shuai Yang, Xiang-Liang Liu, Xiang-Ling Guo, Bin Song.

**Writing – review and editing:** Shuai Yang, Shou-Zhen Li, Ye Feng.
